# Genetically Encoded Copper-Free Click Chemistry**

**DOI:** 10.1002/anie.201008178

**Published:** 2011-03-23

**Authors:** Tilman Plass, Sigrid Milles, Christine Koehler, Carsten Schultz, Edward A Lemke

**Affiliations:** Structural and Computational Biology Unit and Cell Biology and Biophysics Unit, EMBLMeyerhofstrasse 1, 69117 Heidelberg (Germany), Fax: (+49) 6221-397-536

**Keywords:** amino acids, click chemistry, gene expression, proteins, strained molecules

The ability to visualize biomolecules within living specimen by engineered fluorescence tags has become a major tool in modern biotechnology and cell biology. Encoding fusion proteins with comparatively large fluorescent proteins (FPs) as originally developed by the Chalfie and Tsien groups is currently the most widely applied technique.[1] As synthetic dyes typically offer better photophysical properties than FPs, alternative strategies have been developed based on genetically encoding unique tags such as Halo and SNAP tags, which offer high specificity but are still fairly large.[2] Small tags like multi-histidine[3] or multi-cysteine motifs[4] may be used to recognize smaller fluorophores, but within the cellular environment they frequently suffer from poor specificity as their basic recognition element is built from native amino acid side chains. Such drawbacks may be overcome by utilizing bioorthogonal chemistry that relies on coupling exogenous moieties of non-biological origin under mild physiological conditions. A powerful chemistry that fulfils these requirements is the Huisgen type (3+2) cycloaddition between azides and alkynes (a form of click chemistry[5]). By utilizing supplementation-based incorporation techniques and click reactions Beatty et al. coupled azide derivatized dyes to *Escherichia coli* expressing proteins bearing linear alkynes.[6] However, this azide–alkyne cycloaddition required copper(I) as a catalyst (CuAAC), which strongly reduces biocompatibility (but see Ref. [7]). This limitation has been overcome by Bertozzi and co-workers, who showed that the “click” reaction readily proceeds when utilizing ring-strained alkynes as a substrate[8] and since then this strain-promoted azide–alkyne cycloaddition (SPAAC) has found increasing applications in labeling, for example, carbohydrates,[9] nucleotides,[10] and lipids.[11] Further expanding the potential of this approach, Ting and co-workers engineered a lipolic acid ligase which ligates a small genetically encoded recognition peptide to a cylcooctyne-containing substrate. In a second step the incorporated cyclooctyne moiety then functioned as a specific site for labeling in cells.[12]

The direct genetic encoding of fluorescent unnatural amino acids (UAAs) has overcome many drawbacks of previous approaches by offering exquisite specificity, freedom of placement within the target protein, and minimal, if any, structural change. This approach was first achieved by Summerer et al. who evolved a leucyl tRNA/synthetase (tRNA/RS) pair from *E. coli* to genetically encode the UAA dansylalanine into *Saccharomyces cerevisiae*.[13] In response to the amber stop codon TAG, dansylalanine was readily incorporated into proteins by the host translational machinery. This approach has since been used to genetically encode several small dyes,[14] but owing to the need to evolve new tRNA/RS pairs and potential size limitations imposed by the translational machinery, larger dyes with enhanced photophysical properties have not yet been encoded.

**Scheme 1:**
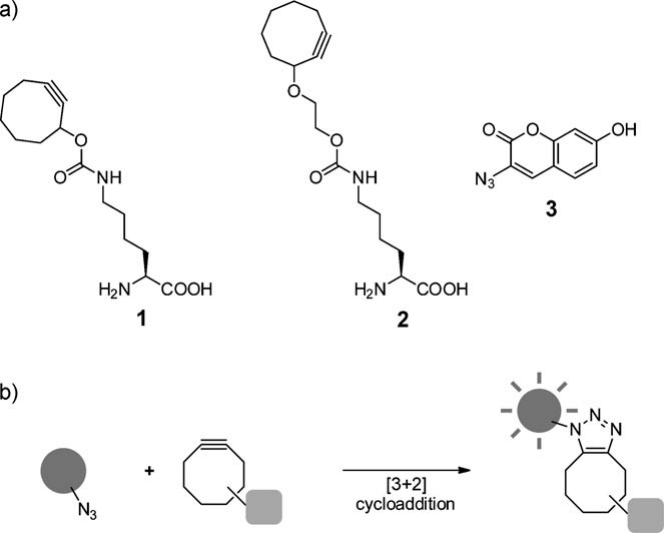
a) Structures of UAAs **1** and **2**, and fluorogenic dye **3**. b) Reaction scheme for copper-free click reaction with fluorogenic azides.

Targeted incorporation of UAAs should in principle make it possible to genetically incorporate strained alkyne and azide functional groups. In fact, a variety of azides have been genetically encoded by the use of, for example, engineered tyrosyl *Methanococcus jannaschii* tRNA^Tyr^/RS, leucyl *E. coli* tRNA^Leu^/RS, and pyrrolysine *Methanosarcina bakeri/mazei* tRNA^pyl^/pylRS[15, 16] pairs (Tyr=tyrosine, Leu=leucyl, pyl=pyrrolysyl). However, genetically encoding the functionality necessary for metal-free click ligations, that is, the strained alkyne, has not been achieved to date, owing to the large size of the cyclic side-chain moiety. Direct encoding of strained alkynes offers numerous advantages over encoding the azide functionality. Many commercial compounds, such as fluorescent dyes or fatty acids, are only available as the azide derivatives and thus not compatible with a copper-free click reaction with the available genetically encoded UAAs. Another major advantage of directly encoding strained alkynes is that they can react with fluorogenic azides, which are dyes whose fluorescence is dramatically quenched in the azide form and increases strongly after a successful click reaction (Scheme 1).[17] This fluorogenic approach was applied, for example, to specifically label lipids within living mammalian cells with a coumarin azide derivative, which offered high contrast due to a lack of background fluorescence.[11] The benefit of fluorogenic labels has also been highlighted in recent work of the Weissleder group who utilized the ability of tetrazines to quench fluorescence and to react with strained dienophiles in a Diels–Alder cycloaddition.[18]

**Figure 1:**
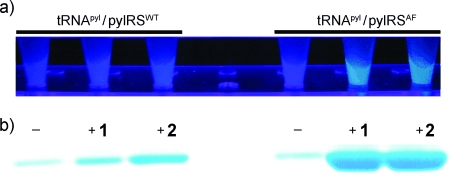
a) Fluorescent images of *E. coli* suspensions in microcentrifuge tubes expressing GFP^TAG^ in the absence (−) and presence (+) of **1** and **2**. b) Corresponding Coomassie stained SDS PAGE gel after purification of GFP^TAG^ by a C-terminal affinity handle.

To overcome the current limitations of engineering copper-free click chemistry into living cells, we aimed to genetically encode strained alkynes into *E. coli*. We reasoned that the natural amber suppressor pyrrolysine tRNA^pyl^/pylRS pair from *M. mazei* might be a suitable starting point because the amino acid substrate binding pocket is partially exposed, offering space to harbor a larger side chain. Furthermore, the carbamate bond at the lysine side chain was recently shown to be an important discriminator for successful incorporation of UAAs.[16, 19, 20] Accordingly, we synthesized lysine derivative **1** in six steps, starting from a commercially available cycloheptene precursor as outlined in Scheme S1 of the Supporting information. In the last step, the *tert*-butoxycabonyl (Boc) protected **1** required particularly mild deprotection with 70 % formic acid, as other standard conditions resulted in cleavage of the side-chain cylcooctyne moiety. The average yield per step was above 80 % and allowed convenient synthesis of gram quantities. We then tested the amber suppression efficiency of the wildtype (WT) system tRNA^pyl^/pylRS^WT^ by co-expressing a green fluorescent protein (GFP) reporter construct. This construct contains an amber TAG mutation at the amino acid position 39 (GFP^TAG^). Hence, full-length and fluorescent GFP is only obtained if the amber stop codon is suppressed. Compared to a control, very little protein expression was observed in the presence of 1 mm **1** as shown by sodium dodecyl sulfate polyacrylamide gel electrophoresis (SDS PAGE) analysis and the fluorescence of the *E. coli* culture (Figure 1 and Figure S1 in the Supporting Information). We then speculated that the proximity of the large side chain to the carbamate bond inhibited efficient substrate recognition by the synthetase. We therefore prepared amino acid **2**, featuring a longer linker between the carbamate and the cyclooctyne group. As evident from Figure 1, a minor improvement of expression yield could be detected. To further improve the acceptance of the UAA, we expanded the substrate binding pocket of pylRS^WT^ by introducing a Y306A mutation into the synthetase and another mutation Y384F, which was previously found by Yanagisawa et al. to support recognition of bulkier side chains.[20] As summarized in Figure 1, GFP^TAG^ was efficiently expressed only in the presence of either **1** or **2** for this mutant tRNA^pyl^/pylRS^AF^ pair and mass spectrometry confirmed the site-specific incorporation of the UAA into the protein (Supporting Information, Table S1). As scaling up the synthesis of **1** was easier than of **2**, we focused on compound **1** for further experiments and determined that typically more than 10 mg GFP^TAG→**1**^ was obtained from a 1 L culture in the presence of 1 mm **1**, demonstrating high suppression efficiency and fidelity of our tRNA^pyl^/pylRS^AF^ construct.

**Figure 2:**
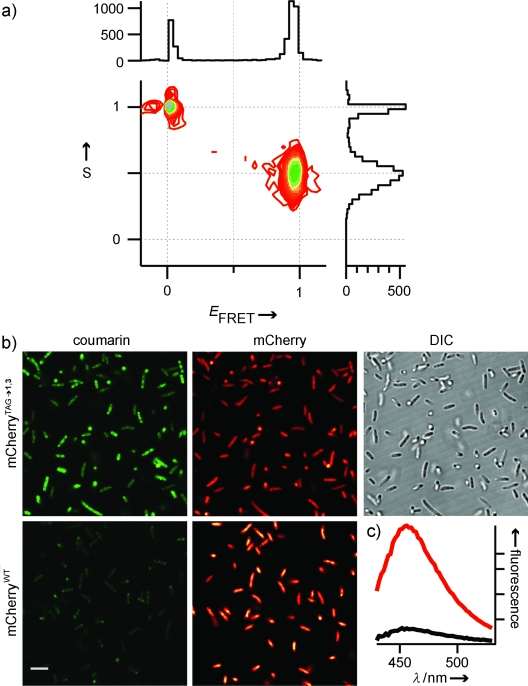
a) 2D histogram (S versus *E_FRET_*) of smFRET data of GFP^TAG→1,Atto647N^ molecules. b) Cells incubated with **3** expressing tRNA^pyl^/pylRS^AF^ with mCherry^TAG→1,3^ (plus differential interference contrast (DIC) image) and mCherry^WT^, imaged for fluorescence of coumarin and mCherry. c) Emission scan for coumarin of *E. coli* suspensions used in (b), mCherry^TAG→1,3^=red, mCherry^WT^=black. Scale bar=5 μm.

To investigate the utility of **1**, we aimed to perform single-molecule (sm) studies of labeled proteins. In particular, single-molecule observation of fluorescence resonance energy transfer (FRET) is a widely applied tool to study protein structure and dynamics. smFRET typically requires covalent, high-yielding, site-specific labeling of proteins with photostable dyes because of the low concentrations used for single-molecule studies and the high demand for overcoming the fundamental noise limit. To this end, we purified GFP^TAG→**1**^ and ligated the commercially available azide derivative of Atto647N under mild conditions. Owing to the spectral properties of this fluorescent species, the natural GFP chromophore served as a donor (D) while Atto647N served as the acceptor dye (A). We then observed freely diffusing GFP^TAG→**1**,Atto647N^ at single-molecule resolution using a confocal detection geometry (Supporting information, Figure S2).[21] Emission bursts stemming from individual GFP^TAG→**1**,Atto647N^ molecules were analyzed based on their labeling stoichiometry (S) and for the occurrence of energy transfer (*E*_FRET_). As shown in Figure 2a, we clearly observed a species that contains both D and A molecules (S=0.5, *E*_FRET_=1). This observed high FRET efficiency is in good agreement with the crystal structure of GFP,[22] indicating that the dye attached at position 39 is within 30 Å of the GFP chromophore.

As mentioned above, the cyclooctyne moiety is ideally suited to achieve site-specific labeling within the living cell. To verify the feasibility of such experiments with our approach, we expressed the red fluorescent protein mCherry^TAG^ in *E. coli* in the presence of 1 mm **1** and tRNA^pyl^/pylRS^AF^. Cells were then incubated in a 50 μm solution of the fluorogenic coumarin azide **3**.[17] Figure 2 shows that only cells expressing mCherry^TAG→**1,3**^ exhibit a strong fluorescence when excited at *λ*=405 nm. The fluorogenic in vivo labeling was also confirmed by fluorescence spectrometry (Figure 2c) and by analyzing the fluorescence of cell lysate using SDS PAGE (see Supporting Information Figure S3–S5, also for additional imaging experiments with other proteins).

In summary, we have genetically encoded one of the most potent functional groups for in vivo chemistry into *E. coli* and demonstrated its basic utility for in vivo labeling as well as high-resolution single-molecule measurements. SPAAC chemistry is now available to site-specifically and non-invasively modify proteins in living cells. As the tRNA^pyl^/pylRS^AF^ showed no obvious dependence on linker length (**1** versus **2**), it is conceivable that slightly altered derivatives, such as mono- and difluorinated cyclooctynes, and possibly bicyclonones,[23] could be directly used in this system. Other enhanced cyclooctynes, such as dibenzocycloctynes,[24] could pose substantial challenges to the synthetase and/or the host translational machinery owing to their larger size. As pylRS from *M. mazei* is orthogonal in a variety of eukaryotic organisms,[19, 25] we are now evaluating the transfer of this system to mammalian cells, where the technique would not only greatly expand our abilities to track proteins in living specimen but also to introduce other type of functional groups, such as cross-linkers or spin-labels for NMR spectroscopy and magnetic resonance imaging (MRI) in living specimens.
