# Supplementary material for: Genetically Encoded Copper-Free Click Chemistry
Source: Angew Chem Int Ed Engl. 2011 Mar 23;50(17):3878–81. doi: 10.1002/anie.201008178 (PMC3210829; doi:10.1002/anie.201008178)
Supplement: Supplementary file 1 [file anie0050-3878-SD1.pdf]

Eine Zeitschrift der Gesellschaft Deutscher Chemiker

Supporting Information

© Wiley-VCH 2011

69451 Weinheim, Germany

# Genetically Encoded Copper-Free Click Chemistry**

*Tilman Plass, Sigrid Milles, Christine Koehler, Carsten Schultz,* and Edward A. Lemke**

anie_201008178_sm_miscellaneous_information.pdf

## Supporting information

### Material and methods

### General methods

Unless otherwise noted, materials for chemical synthesis were obtained from commercial suppliers (Sigma-Aldrich, Aldrich, Sigma, Fluka, Acros, Iris) in the highest purity available and used without further purification. Dry solvents were purchased from Sigma-Aldrich, Acros, and Fluka, stored over molecular sieves and used as supplied. Solvents used for extraction and chromatography were purchased from Fluka, Thermo Fisher Scientific, Merck, and BDH Prolabo (VWR). Flash chromatography (FC) was carried out using Merck silica gel 60 (63-200 mesh). Thin layer chromatography (TLC) was performed on aluminium-backed, precoated silica gel plates (Macherey-Nagel Alugram Sil G/$UV_{254}$ and Merck silica gel 60 $WF_{254s}$) with cHex/EtOAc or DCM/MeOH/AcOH mixtures as mobile phases. Spots were detected by a UV hand lamp at λ= 254 nm or λ= 366 nm or staining with either a) anisaldehyde staining solution (85 ml EtOH, 10 ml AcOH, 5 ml concentrated $H_2SO_4$, 0.5 ml anisaldehyde), b) $KMnO_4$ staining solution (3.0 g $KMnO_4$, 20 g $K_2CO_3$ in 300 ml 5% aqueous NaOH), or c) ninhydrin staining solution (250 ml EtOH, 1.5 ml AcOH, 0.5 g ninhydrin) and subsequent heat treatment.

NMR spectra were recorded using a Bruker UltraShieldTM Advance 400 (400 MHz, $^1$H; 100 MHz, $^{13}$C) spectrometer and calibrated using residual undeuterated solvent as an internal reference. High-resolution (HR) mass spectra were recorded at the University of Heidelberg using electrospray ionization (ESI) MS on a Bruker ApexQe hybrid 9.4 T FT-ICR mass spectrometer. Products were characterized by NMR ($^1$H, $^{13}$C), and HR MS.

### Plasmids and DNA constructs:

An *E. coli* codon optimized gene for *M. mazei* $tRNA^{pyl}$/$pylRS^{WT}$ was purchased from Mr Gene (Regensburg, Germany). The pEvol plasmid system,[1] kindly provided by Peter G. Schultz (The Scripps Research Institute, La Jolla, CA), served as a starting point to replace the two coding regions for the *M. jannaschii* RS in these plasmids by the optimized codon $tRNA^{pyl}$/$pylRS^{WT}$ as well as the tRNA, which generated the plasmid pEvol $tRNA^{pyl}$/$pylRS^{WT}$ used in this work. For the double mutant, two rounds of standard site-directed mutagenesis were performed to introduce Y306A and Y384F into the codon optimized gene. As for the wildtype (WT), two copies of this gene were then cloned into the pEvol plasmid to generate the mutant plasmid pEvol $tRNA^{pyl}$/$pylRS^{AF.}$

### Protein expression and purification:

For expression control, a pBAD (Invitrogen, Carlsbad, USA) plasmid harbouring an N-terminal FLAG tagged GFP with a C-terminal 6-his peptide sequence was used, where the permissive site 39 contained the amber (TAG) stop codon. Analogously, also an N-terminal FLAG tagged mCherry with a C-terminal 6-his peptide sequence was used, where a permissive site in the linker region between the FLAG sequence and mCherry contained the Amber (TAG) stop codon. Both plasmids were co-transformed into *E. coli* Top10 cells (Invitrogen) grown at 37°C in the presence of ampicillin and chloramphenicol. For small scale expression, typically 0.5 ml of an overnight culture was used to inocculate 50 ml Terrific Broth medium in a shake flask. Cultures typically grew within 1-2 h to an $OD_{600}$ of 0.2-0.3 at which point compound **1** or **2** (stock solution 80 mM in 0.1 M NaOH) or an equal amount of 0.1 M NaOH (for control experiments) were added to a final concentration of 1 mM **1** or **2**. The cultures were allowed to grow until an $OD_{600}$ of 04.-0.6, until expression was induced with 0.02% arabinose. Cultures were harvested by centrifugation after 4-6 h of shaking at 37°C by centrifugation. Pellets were resuspended in a 4x phosphate buffered saline (PBS, pH 8.0) solution, and cells were lyzed by sonication. The lysate was centrifuged for 1 h at 14000xg and the supernatant was incubated with ~50 µl of Ni-NTA agarose (Qiagen, Dusseldorf, Germany). Beads were washed with 10 mM imidazole in 4x PBS and then eluted with buffer containing 500 mM imidazole. Wherever mentioned, washing and/or elution was also carried out in a 4x PBS denaturing buffer containing 6 M guanidinium hydrochloride (GdmCl). Larger expressions, used to determine yields more accurately, were scaled up accordingly.

### Single molecule spectroscopy

A 50 µM to 1 mM solution of $GFP^{TAG\rightarrow\mathbf{1}}$ in 4x PBS (pH 8.0) was incubated for 12 h at 37°C with a moderate excess (10x) of the commercially available Atto647N azide (Atto-Tec GmbH, Siegen, Germany). The mixture was incubated with Ni-NTA agarose and washed with 2 M Urea 4x PBS (mildly denaturing) to remove any nonspecifically bound dye from the protein and then eluted in native 4x PBS buffer to induce refolding. Typical labelling efficiencies were about 50%, as determined using standard UV/Vis spectrometry and the reported extinction coefficients for GFP, denatured GFP (if measured under denaturing conditions) and Atto647N.

### Ensemble spectroscopy

Ensemble fluorescence spectroscopy measurements were performed on a PTI Quantamaster spectrometer (Ontario, Canada).

GFP$^{TAG\rightarrow\mathbf{1},Atto647N}$ was diluted to 50 pM and analysed via single molecule spectrometry of freely diffusing molecules similar to previously reported measurements schemes.[2] Briefly, the solution was mounted on a custom built confocal microscope centered around a Olympus IX81 microscope (Hamburg, Germany) equipped with a 1.2NA 60x water objective. The light emitting from two laserdiodes (LDH 485 and 660, Picoquant, Berlin, Germany) was alternated at a master pulse frequency of 50 MHz and focused into the sample. The burst wise fluorescence light emitting from single, freely diffusing GFP$^{TAG\rightarrow\mathbf{1},Atto647N}$ was spatially filtered using a 100 µm pinhole and then spectrally filtered into fluorescent donor (D) and acceptor (A) channels. (using emission filters 525/50, 700/75, dichroics 497/661 and 555 from AHF, Tuebingen Germany). Single photons were detected using MPDs (Picoquant) in the green and APDs (PerkinElmer, Vaudreuil Canada) in the red channel. Signals were counted using a Hydraharp (Picoquant) and subject to routine pulsed interleaved excitation analysis[3] after binning the signal stream to a 1 ms bin width and applying a threshold of 100 counts per single molecule burst (see also Figure S2). This makes it possible to determine the labelling stoichiometry (S) of the samples as well the efficiency of fluorescence energy transfer ($E_{FRET}$) from D to A. Figure 2a) shows such a two dimensional S vs $E_{FRET}$ plot. Since the Atto647N dye attachment site (due to its known spectral properties here serving as A is within 30 Å of the chromophore of the GFP (D) we expect a high $E_{FRET}$. As expected we can observe two populations. One population is centered around S= 1 and $E_{FRET}$= 0, which corresponds to molecules with D only. This population is almost always observed in single molecule experiments (see [2]) and originates from those species where A was either photophysically inactive or not present. The second population is centered around S= 0.5 and $E_{FRET}$= 1 and clearly identifies a population of GFP labelled with Atto647N so that energy transfer occurs efficiently.

**Compound synthesis**

The synthesis of compound **1** and **2** are summarized in Scheme S1 and S2.

**Scheme S1:** Synthesis of cyclooctynyl lysine derivative **1**. Reagents and conditions: a) TEA, THF, –10˚C to RT, 83%; b) Boc-L-Lys-OH, TEA, DMF, 0°C to RT, 91%; c) formic acid, $CHCl_3$, RT, 96%. TEA= triethylamine; THF= tetrahydrofuran; RT= room temperature; Boc-L-Lys-OH= *N*-*α*-*t*-butyloxycarbonyl-L-lysine; DMF= dimethylformamide. The overall yield after six steps was 37%.

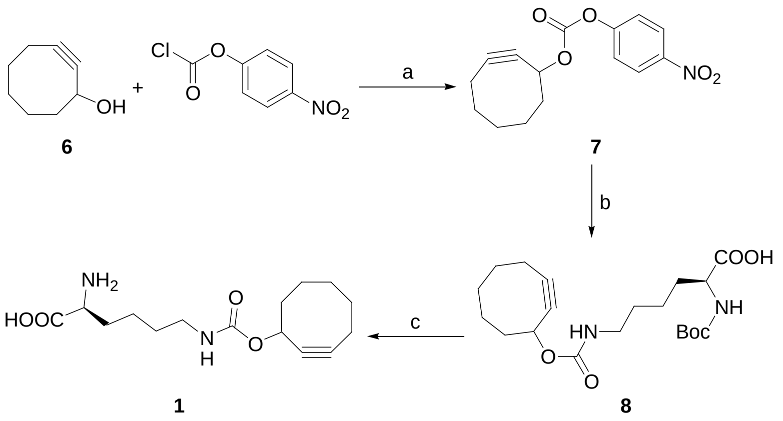


**Scheme S2:** Synthesis of cyclooctynyl lysine derivative **2**. Reagents and conditions: a) $AgClO_4$, acetone, RT, dark, 49%; b) DBU, DMSO, 60 °C, 74%; c) TEA, THF, –10°C to RT, 65%; d) Boc-L-Lys-OH, TEA, DMF, 0°C to RT, 79%; e) formic acid, $CHCl_3$, RT, 94%. RT= room temperature; DBU= 1,8-diazabicyclo[5.4.0]undec-7-ene; DMSO= dimethylsulfoxide; TEA= triethylamine; THF= tetrahydrofuran; Boc-L-Lys-OH= *N*-*α*-*t*-butyloxycarbonyl-L-lysine; DMF= dimethylformamide.

Occasionally, we observed step **a** to result in low yields of **9** giving **5** as a side product, likely due to high water sensitivity of this step. In addition, the overall yield after six steps was 11% (compare to 37% for **1**) and consequently, our applications focused on utilizing **1.**

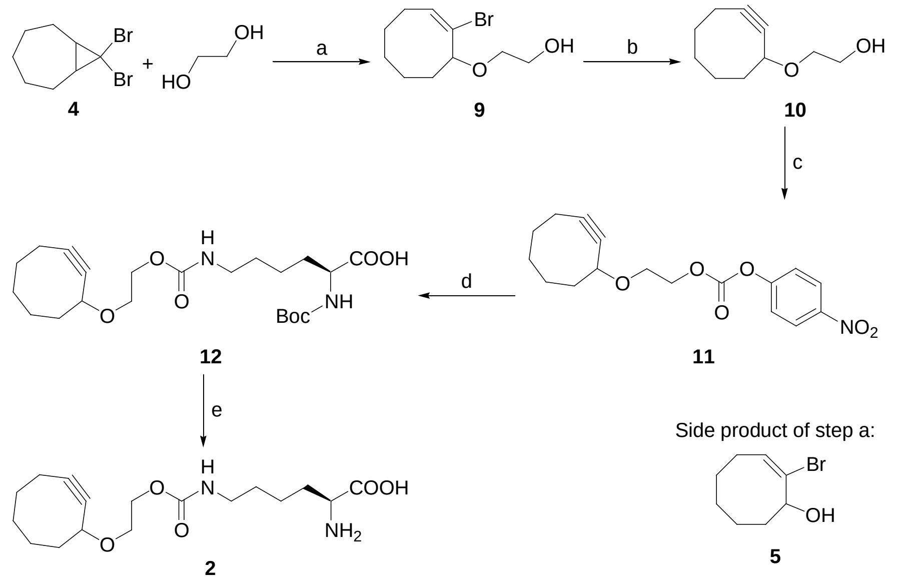


Literature known 8,8-Dibromobicyclo[5.1.0]octane **4**[4] was synthesized starting from commercially available *cis*-cycloheptene as reported earlier. 2-Bromocyclooct-2-en-1-ol **5**[5] and cyclooct-2-yn-1-ol **6**[6] were synthesized according to previously described protocols. Coumarin azide **3** was synthesized according to published protocols.[7]

**Cyclooct-2-yn-1-yl 4-nitrophenyl carbonate (7)**

Compound **6** (3.12 g, 25.1 mmol) and TEA (4.20 ml, 30.2 mmol, 1.2 eq) were dissolved in THF (0.2 M, 126 ml) and added dropwise to a stirred solution of 4-nitrophenyl chloroformate (15.2 g, 75.4 mmol, 3.0 eq) and THF (0.7 M, 36 ml) over a period of 1 h at –10°C. The reaction mixture was allowed to warm up to RT and was stirred overnight. Then cHex (100 ml) was added and the THF was removed under reduced pressure. Filtration of the reaction mixture followed by FC (cHex:EtOAc 9:1) gave **7** as a yellow oil (6.03 g, 20.9 mmol, 83%).

$R_f$ (cHex/EtOAc 4:1)= 0.70.

$^1$H-NMR ($CDCl_3$) **δ=** 8.28 (dt, $^3J$ = 9.30, $^3J$ = 2.72, 2H, $CH^{aromatic}$), 7.40 (dt, $^3J$ = 9.30, $^3J$ = 2.72, 2H, $CH^{aromatic}$), 5.30-5.35 (m, 1H, $CH^{propargyl}$), 2.10-2.38 (m, 3H, $CH_2^{ring}$), 1.92-1.99 (m, 2H, $CH_2^{ring}$), 1.74-1.88 (m, 3H, $CH_2^{ring}$), 1.59-1.64 (m, 2H, $CH_2^{ring}$) ppm.

$^{13}$C-NMR ($CDCl_3$) **δ=** 155.5 (*C*(O)$O_2$), 155.1, 151.5 (2× $C^{aromatic}$), 125.5, 121.8 (2× $CH^{aromatic}$), 104.1, 89.2 (2× $C^{ring}$), 72.0 ($CH^{propargyl}$), 41.4, 34.1, 29.4, 25.9, 20.7 (5× $CH_2^{ring}$) ppm.

***N*-*α*-*tert*-Butyloxycarbonyl-*N*-*ε*-(cyclooct-2-yn-1-yloxy)carbonyl)-L-lysine (8)**

Compound **7** (0.93 g, 3.21 mmol) was dissolved in DMF (0.2 M, 16 ml) and added dropwise to a stirred solution of Boc-L-Lys-OH (1.03 g, 4.18 mmol, 1.3 eq) and TEA (1.35 ml, 9.64 mmol, 3.0 eq) in DMF (0.5 M, 6 ml) over a period of 1 h at 0°C. The reaction mixture was stirred at RT overnight. After removal of all volatile components by evaporation under reduced pressure, the residue was taken up in $H_2O$ (100 ml) and EtOAc (100 ml). The aqueous phase was acidified with concentrated HCl and extracted with EtOAc (3× 50 ml). The combined organic layers were washed with saturated NaCl solution and dried over $Na_2SO_4$. The solvent was evaporated under reduced pressure and the crude product was purified by FC (DCM/MeOH/AcOH 97:2:1) to yield **8** as a very viscous yellow oil (1.17 g, 2.94 mmol, 91%).

$R_f$ (DCM/MeOH/AcOH 97:2:1)= 0.45.

$^1$H-NMR ($CDCl_3$) **δ=** 5.26-5.32 (m, 1H, $CH^{propargyl}$), 5.19-5.25 (m, 1H, N*H*), 4.80-4.86 (m, 1H, N*H*), 4.24-4.34 (m, 1H, *α*-$CH^{Lys}$), 3.16 (q, $^3J$ = 6.32, 2H, *ε*-$CH_2^{Lys}$), 2.10-2.31 (m, 3H, $CH_2^{ring}$), 1.63-2.04 (m, 9H, $CH_2^{ring}$, $CH_2^{Lys}$), 1.49-1.57 (m, 4H, $CH_2^{Lys}$), 1.45 (s, 9H, Boc) ppm.

$^{13}$C-NMR ($CDCl_3$) **δ=** 176.5 (*C*(O)$O_2$), 156.1 ($C^{Lys}$), 154.7 ($C^{Boc}$), 101.6, 91.2 (2× $C^{ring}$), 80.1 ($C(CH_3)_3^{Boc}$), 67.1 ($CH^{propargyl}$), 53.3 (*α*-$CH^{Lys}$), 41.9 ($CH_2^{ring}$), 40.5 (*ε*-$CH_2^{Lys}$), 34.2 ($CH_2^{ring}$), 31.9 ($CH_2^{Lys}$), 29.7 ($CH_2^{ring}$), 29.3 ($CH_2^{Lys}$), 28.5 (3× $CH_3^{Boc}$ ), 26.2 ($CH_2^{ring}$), 22.4 ($CH_2^{Lys}$), 20.7 ($CH_2^{ring}$) ppm.

**N-ε-(Cyclooct-2-yn-1-yloxy)carbonyl)-L-lysine (1)**

Compound **8** (1.24 g, 3.13 mmol) was dissolved in 70% formic acid in $CHCl_3$ (0.2 M, 16 ml) and stirred for 36 h at RT. DMF (0.2 M, 16 ml) was added and all volatile components were removed under reduced pressure. The residual was taken up in 0.1 M HCl (100 ml) and lyophilized affording the pure HCl salt of **1** as a faint yellow solid (1.00 g, 3.01 mmol, 96%).

$^1$H-NMR (DMSO-d$_6$) δ= 7.43-7.79 (m, 2H, $\alpha$-N$H_2$), 7.18 (t, $^3J$ = 5.78, 1H, $\varepsilon$-N$H$), 5.09-5.13 (m, 1H, C$H^{propargyl}$), 3.05 (t, $^3J$ = 5.72, 1H, $\alpha$-C$H^{Lys}$), 2.90 (q, $^3J$ = 5.78, 2H, $\varepsilon$-C$H_2^{Lys}$), 2.00-2.26 (m, 3H, C$H_2^{ring}$), 1.77-1.91 (m, 3H, C$H_2^{ring}$), 1.63-1.71 (m, 2H, C$H_2^{ring}$), 1.41-1.59 (m, 4H, C$H_2^{ring}$, C$H_2^{Lys}$), 1.23-1.37 (m, 4H, C$H_2^{Lys}$) ppm.

$^{13}$C-NMR (DMSO-d$_6$) δ= 170.8 ($C$(O)O$_2$), 155.7 ($C^{Lys}$), 101.2, 92.4 (2× $C^{ring}$), 66.0 ($C$H$^{propargyl}$), 54.4 ($\alpha$-$C$H$^{Lys}$), 42.1 ($C$H$_2^{ring}$), 40.6 ($\varepsilon$-$C$H$_2^{Lys}$), 34.3 ($C$H$_2^{ring}$), 31.1 ($C$H$_2^{Lys}$), 29.7 ($C$H$_2^{ring}$), 29.5 ($C$H$_2^{Lys}$), 26.3 ($C$H$_2^{ring}$), 22.8 ($C$H$_2^{Lys}$), 20.4 ($C$H$_2^{ring}$) ppm.

HR-ESI MS: $[M+H]^+$ calculated: 297.18088, $[M+H]^+$ found: 297.18083; $[M+Na]^+$ calculated: 319.16283, $[M+Na]^+$ found: 319.16282; $[M+K]^+$ calculated: 335.13677, $[M+K]^+$ found: 335.13679.

**2-(Bromocyclooct-2-en-1-yloxy)ethanol (9)**

Compound **4** (3.12 g, 11.6 mmol) and anhydrous ethane-1,2-diol (13.0 ml, 23.3 mmol, 20.0 eq) were dissolved in anhydrous acetone (0.6 M, 19 ml). Anhydrous $AgClO_4$ (7.24 g, 34.9 mmol, 3.0 eq) was added in small portions under exclusion of light and stirred at RT for 1 h. After addition of EtOAc (100 ml) and filtration, 1 M HCl (100 ml) was added and the aqueous layer was extracted with EtOAc (3× 50 ml). The combined organic layers were washed with 1 M HCl/$H_2O$/saturated NaCl solution (100 ml each) and dried over $Na_2SO_4$. The solvent was evaporated under reduced pressure and **9** (1.42 g, 5.68 mmol, 49%) was obtained as a yellow oil and used without further purification.

$^1$H-NMR (CDCl$_3$) δ= 6.20 (dd, $^3J$ = 11.73, $^3J$ = 4.09, 1H, C$H^{vinyl}$), 3.91 (dd, $^3J$ = 10.22, $^3J$ = 5.09, 1H, C$H^{allyl}$), 3.78 (t, $^3J$ = 4.51 2H, C$H_2^{ethyl}$), 3.61-3.66 (m, 1H, C$H$H'$^{ethyl}$), 3.44-3.49 (m, 1H, CH$H'^{ethyl}$), 2.27-2.34 (m, 2H, C$H_2^{ring}$), 1.84-2.06 (m, 2H, C$H_2^{ring}$), 1.67-1.76 (m, 2H, C$H_2^{ring}$), 1.43-1.55 (m, 2H, C$H_2^{ring}$), 1.23-1.34 (m, 2H, C$H_2^{ring}$) ppm.

$^{13}$C-NMR (CDCl$_3$) δ= 132.8 ($C$Br), 131.7 ($C$H$^{vinyl}$ ), 85.0 ($C$H$^{allyl}$), 69.9, 61.9 (2× $C$H$_2^{ethyl}$), 39.6, 36.5, 33.3, 28.1, 26.3 (5× $C$H$_2^{ring}$) ppm.

HR-ESI MS: $[M+Na]^+$ calculated: 271.03041, $[M+Na]^+$ found: 271.03046; $[M+K]^+$ calculated: 287.00435, $[M+K]^+$ found: 287.00443.

**2-(Cyclooct-2-yn-1-yloxy)ethanol (10)**

Compound **9** (3.76 g, 15.1 mmol) was dissolved in DMSO (0.5 M, 30 ml) and heated to 60˚C. DBU (4.51 ml, 30.2 mmol, 2.0 eq) was added, the resulting solution was stirred for 15 min and more DBU (18.0 ml, 121 mmol, 8.0 eq) was added. The mixture was stirred at 60˚C overnight and then cooled to RT. EtOAc (100 ml) and water (100 ml) were added. After acidification to pH 1 with concentrated HCl, the aqueous phase was extracted with EtOAc (3× 50 ml). The combined organic layers were washed with 1 M HCl/saturated NaCl solution (100 ml each), dried over $Na_2SO_4$ and evaporated under reduced pressure. FC (cHex/EtOAc 9:1) afforded **10** (1.89 g, 11.2 mmol, 74%) as a light yellow oil.

$R_f$ (cHex/EtOAc 4:1)= 0.24.

$^1$H-NMR (CDCl$_3$) δ= 4.20-4.24 (m, 1H, C$H^{propargyl}$), 3.72-3.77 (m, 2H, C$H_2^{ethyl}$), 3.65-3.71 (m, 1H, C$H$H'$^{ethyl}$), 3.44-3.50 (m, 1H, CH$H'^{ethyl}$), 2.10-2.31 (m, 3H, C$H_2^{ring}$), 1.91-2.03 (m, 2H, C$H_2^{ring}$), 1.78-1.89 (m, 2H, C$H_2^{ring}$), 1.58-1.74 (m, 2H, C$H_2^{ring}$), 1.42-1.51 (m, 1H, C$H_2^{ring}$) ppm.

$^{13}$C-NMR (CDCl$_3$) δ= 100.5, 92.5 (2× $C^{ring}$), 72.8 ($C$H$^{propargyl}$), 70.4, 61.9 (2× $C$H$_2^{ethyl}$), 42.3, 34.3, 29.8, 26.3, 20.7 (5× $C$H$_2^{ring}$) ppm.

**2-(Cyclooct-2-yn-1-yloxy)ethyl 4-nitrophenyl carbonate (11)**

Compound **10** (2.64 g, 15.7 mmol) and TEA (2.63 ml, 18.8 mmol, 1.2 eq) dissolved in THF (0.2 M, 79 ml) were added dropwise to a stirred solution of 4-nitrophenyl chloroformate (9.49 g, 47.1 mmol, 3.0 eq) and THF (0.7 M, 22 ml) over a period of 1 h at –10°C. The reaction mixture was stirred overnight at RT and diluted with cHex (100 ml). Then cHex (100 ml) was added and the THF was removed under reduced pressure. Filtration of the reaction mixture followed by FC (cHex:EtOAc 4:1) gave **11** as a yellow oil (3.42 g, 10.3 mmol, 65%).

$R_f$ (cHex/EtOAc 4:1) = 0.44.

$^1$H-NMR ($CDCl_3$) δ = 8.28 (dt, $^3J$ = 9.18, $^3J$ = 2.12, 2H, C$H^{aromatic}$), 7.39 (dt, $^3J$ = 9.18, $^3J$ = 2.12, 2H, C$H^{aromatic}$), 4.41-4.47 (m, 2H, C$H_2^{ethyl}$), 4.25-4.30 (m, 1H, C$H^{propargyl}$), 3.86-3.92 (m, 1H, C$H$H'$^{ethyl}$), 3.63-3.69 (m, 1H, CH$H'^{ethyl}$), 2.11-2.32 (m, 3H, C$H_2^{ring}$), 1.91-2.05 (m, 2H, C$H_2^{ring}$), 1.79-1.89 (m, 2H, C$H_2^{ring}$), 1.62-1.73 (m, 2H, C$H_2^{ring}$), 1.42-1.52 (m, 1H, C$H_2^{ring}$) ppm.

$^{13}$C-NMR ($CDCl_3$) δ = 156.5 ($C$(O)$O_2$), 155.6, 152.5 (2× $C^{aromatic}$), 125.3, 122.3 (2× $C$H$^{aromatic}$), 101.0, 92.1 (2× $C^{ring}$), 73.0 ($C$H$^{propargyl}$), 68.5, 66.3 (2× $C$H$_2^{ethyl}$), 42.3, 34.3, 29.7, 26.3, 20.7 (5× $C$H$_2^{ring}$) ppm.

**N-α-tert-Butyloxycarbonyl-N-ε-(2-(cyclooct-2-yn-1-yloxy)ethyl) carbonyl-L-lysine (12)**

Compound **11** (0.60 g, 1.81 mmol) was dissolved in DMF (0.2 M, 9 ml) and added dropwise to a stirred solution Boc-L-Lys-OH (0.58 g, 2.35 mmol, 1.3 eq) and TEA (0.76 ml, 5.42 mmol, 3.0 eq) in DMF (0.5 M, 4 ml) over a period of 1 h at 0°C. The reaction mixture was stirred at RT overnight. After removal of all volatile components by evaporation under reduced pressure, the residue was taken up in $H_2O$ (100 ml) and EtOAc (100 ml). The aqueous phase was acidified with concentrated HCl, and extracted with EtOAc (3× 50 ml). The combined organic layers were washed with saturated NaCl solution and dried over $Na_2SO_4$. The solvent was evaporated under reduced pressure and the crude product was purified by FC (DCM/MeOH/AcOH 97:2:1) to yield **12** as a very viscous yellow oil (0.631 g, 1.43 mmol, 79%).

$R_f$ (DCM/MeOH/AcOH 97:2:1)= 0.41.

$^1$H-NMR ($CDCl_3$) δ= 5.18-5.23 (m, 1H, N$H$), 4.79-4.85 (m, 1H, N$H$), 4.16-4.31 (m, 4H, C$H^{propargyl}$, α-C$H^{Lys}$, C$H_2^{ethyl}$), 3.74-3.81 (m, 1H, C$H$H'$^{ethyl}$), 3.52-3.60 (m, 1H, CH$H'^{ethyl}$), 3.15-3.26 (m, 2H, ε-C$H_2^{Lys}$), 2.11-2.31 (m, 3H, C$H_2^{ring}$), 1.51-2.04 (m, 13H, C$H_2^{ring}$, C$H_2^{Lys}$), 1.45 (s, 9H, Boc) ppm.

$^{13}$C-NMR ($CDCl_3$) δ= 175.8 ($C$(O)$O_2$), 156.7 ($C^{Lys}$), 155.9 ($C^{Boc}$), 100.6, 92.4 (2× $C^{ring}$), 80.2 ($C$($CH_3$)$_3^{Boc}$), 72.7 ($C$H$^{propargyl}$), 67.4, 63.9 (2× $C$H$_2^{ethyl}$), 53.2 (α-$C$H$^{Lys}$), 42.2 ($C$H$_2^{ring}$), 40.5 (ε-$C$H$_2^{Lys}$), 34.3 ($C$H$_2^{ring}$), 31.8 ($C$H$_2^{Lys}$), 29.8 ($C$H$_2^{ring}$), 29.3 ($C$H$_2^{Lys}$), 28.3 (3× $C$H$_3^{Boc}$), 26.4 ($C$H$_2^{ring}$), 22.3 ($C$H$_2^{Lys}$), 20.7 ($C$H$_2^{ring}$) ppm.

HR-ESI MS: $[M+H]^+$ calculated: 441.25953, $[M+H]^+$ found: 441.25982; $[M+Na]^+$ calculated: 463.24147, $[M+Na]^+$ found: 463.24172; $[M+K]^+$ calculated: 479.21541, $[M+K]^+$ found: 479.21570.

**N-ε-(2-(Cyclooct-2-yn-1-yloxy)ethyl) carbonyl-L-lysine (2)**

Compound **12** (2.08 g, 4.71 mmol) was dissolved in 70 % formic acid in $CHCl_3$ (0.2 M, 24 ml) and stirred for 36 h at RT. DMF (0.2 M, 24 ml) was added and all volatile components were removed under reduced pressure. The residual was taken up in 0.1 M HCl (100 ml) and lyophilized, affording the pure HCl salt of **2** as a yellow solid (1.50 g, 4.42 mmol, 94%).

$^1$H-NMR (DMSO-$d_6$) δ= 7.34-7.65 (m, 2H, α-N$H_2$), 7.14-7.23 (m, 1H, ε-N$H$), 4.17-4.25 (m, 1H, C$H^{propargyl}$), 3.95-4.07 (m, 2H, C$H_2^{ethyl}$), 3.49-3.60 (m, 1H, C$H$H'$^{ethyl}$), 3.38-3.43 (m, 1H, CH$H'^{ethyl}$), 3.05 (t, $^3J$ = 6.03, 1H, α-C$H^{Lys}$), 2.92 (q, $^3J$ = 6.23, 2H, ε-C$H_2^{Lys}$), 1.98-2.24 (m, 2H, C$H_2^{ring}$), 1.59-1.86 (m, 5H, C$H_2^{ring}$), 1.44-1.57 (m, 3H, C$H_2^{ring}$, C$H_2^{Lys}$), 1.22-1.39 (m, 6H, C$H_2^{ring}$, C$H_2^{Lys}$) ppm.

$^{13}$C-NMR (DMSO-$d_6$) δ= 171.2 ($C$(O)$O_2$), 156.8 ($C^{Lys}$), 100.4, 93.4 (2× $C^{ring}$), 72.3 ($C$H$^{propargyl}$), 67.5, 65.9 (2× $C$H$_2^{ethyl}$), 53.6 (α-$C$H$^{Lys}$), 42.3 ($C$H$_2^{ring}$), 40.5 (ε-$C$H$_2^{Lys}$), 34.4 ($C$H$_2^{ring}$), 30.7 ($C$H$_2^{Lys}$), 29.8 ($C$H$_2^{Lys}$), 29.6 ($C$H$_2^{ring}$), 26.4 ($C$H$_2^{ring}$), 22.5 ($C$H$_2^{Lys}$), 20.5 ($C$H$_2^{ring}$) ppm.

HR-ESI MS: $[M+H]^+$ calculated: 341.20710, $[M+H]^+$ found: 341.20716; $[M+Na]^+$ calculated: 363.18917, $[M+Na]^+$ found: 363.18904.

**Results**

**Table S1:** For mass spectroscopic validation the bands shown in the Coomassie stained gel Figure S1 for $GFP^{TAG\rightarrow\mathbf{1}}$ and $GFP^{TAG\rightarrow\mathbf{2}}$ expressed in *E. coli* harbouring the $tRNA^{pyl}$/$pylRS^{AF}$ plasmid were excised and subsequently digested with trypsin (tryp) and chymotrypsin (chytryp) following standard protocols for high resolution peptide mass analysis. Peptides were analysed using an Orbitrap mass spectrometer (Thermofisher, USA). The data was analysed using the Mascot algorithm and the results verifying incorporation of **1** and **2** are summarized in the table.

| | Protease | Monoisotopic mass (calc) [Da] | Match mass, found [Da] | Δ Mass [Da] | Ion score | Peptide sequence (X= amber TAG site) |
|---|---|---|---|---|---|---|
| GFP$^{TAG\rightarrow\mathbf{1}}$ | chytryp | 1583.76788 | 1583.76788 | (-)0.00003 | 76 | SVSGEGEGDATXGKL |
| GFP$^{TAG\rightarrow\mathbf{1}}$ | tryp | 1617.75224 | 1617.75224 | (-)0.00005 | 84 | FSVSGEGEGDATXGK |
| GFP$^{TAG\rightarrow\mathbf{2}}$ | chytryp | 1627.79410 | 1627.79410 | (-)0.00007 | 31 | SVSGEGEGDATXGKL |
| GFP$^{TAG\rightarrow\mathbf{2}}$ | tryp | 1661.77846 | 1661.77846 | (-)0.00008 | 39 | FSVSGEGEGDATXGK |

**Figure S1:** Full size SDS, Coomassie stained gel of Figure 1 in the main text. GFP$^{TAG}$ was expressed in the presence of WT or mutant tRNA/RS pairs in media supplemented with either NaOH, **1** or **2** (marker given in kDa).

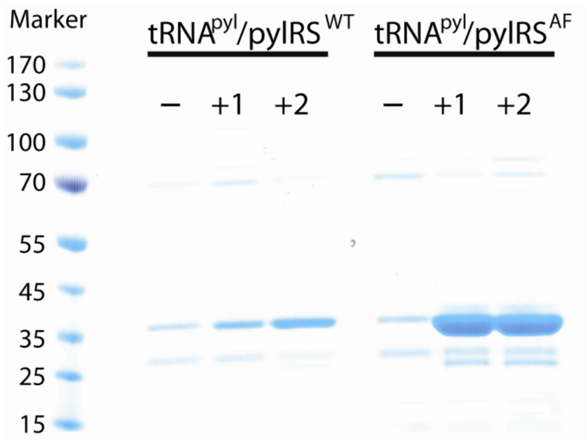


**Figure S2:** Shown is the raw data trace (binned at 1 ms time resolution) of single freely diffusing GFP$^{TAG\rightarrow\mathbf{1},Atto647N}$ corresponding to the data shown in the main text Figure 2. Fluorescent bursts detected in the donor channel (green) stem from the directly excited GFP chromophore (and little or no energy transfer to A) and bursts in the red channel (A) originate from resonance energy transfer to the Atto647N dye. In agreement with destruction of the GFP chromophore, no FRET signal could be observed when denaturing the protein in 6 M GdmCl (and boiling for 5 min at 95°C, data not shown).

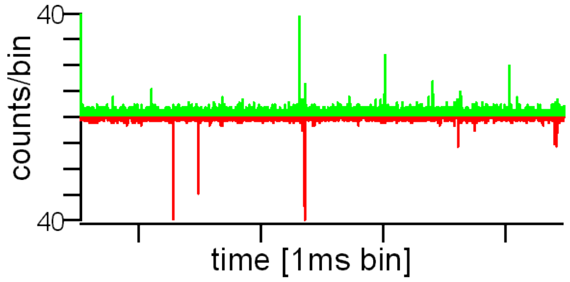


**Figure S3:** For fluorescence imaging of fluorogenic coumarin azide **3**, 5 ml tRNA$^{pyl}$/pylRS$^{AF}$ containing *E. coli* cultures expressing either mCherry$^{TAG\rightarrow\mathbf{1}}$ or mCherry$^{WT}$ were harvested after overnight induction and incubation with **1**, washed 2x with 12 ml PBS, resuspended and incubated in 12 ml PBS for 1 h at 4°C in the dark, and washed another 2x with 12 ml PBS. Cells were pelleted and resuspended in 3 ml PBS ($OD_{600}$ ~2-3) and then incubated with 50 µM **3** at 37˚C shaking in the dark. Cells were harvested after 3-4 h, washed two times with 1.5 ml PBS, resuspended in 1.5 ml PBS and incubated for 1 h at 4°C in the dark, and washed another two times with 1.5 ml PBS before cells were allowed to settle on a coverslip. Cells were then mounted on a Leica SP5 microscope employing a 1.4NA 63x oil objective (Leica, Mannheim, Germany). Images containing 512*512 pixels were acquired at a scan speed of 400 Hz and a zoom factor of three yielding a final pixel size of 160.5 nm*160.5 nm. In addition to a DIC image (e, l) the sample was excited using a blue diode laser operating at a wavelength of 405 nm, while simultaneously recording the fluorescence signal in two channels (blue/green= 420-520 nm and red= 590-690 nm). With cells expressing mCherry$^{TAG\rightarrow\mathbf{1}}$, fluorescence in the blue/green channel originates from clicked coumarin (a), while mCherry in the red channel is not visible when exciting with 405 nm (b). In panel (c, d) the same emission channels were recorded during excitation with a DPSS laser operating at λ= 561 nm, which only excites mCherry. As a control

experiment the same imaging procedure was repeated with cells expressing $mCherry^{WT}$ (and also incubated with **3** as above). In this construct, the synthetase and tRNA are still active in recognizing **1**, but wild type mCherry contains no amber codon that allows incorporation of **1**. Consequently, only background fluorescence is visible in (f). Since $mCherry^{WT}$ naturally expresses better than the amber suppressed $mCherry^{TAG \rightarrow \mathbf{1}}$ a stronger red fluorescence is observed in (k) (compared to (d)), although the PMT gain was reduced in the red channel in addition (k). In contrast to Figure 2b) in the main text, the images shown in this figure are full size images. Scale bar= 5 µm. Contrast/brightness were adjusted for image clarity in the blue/green channel.

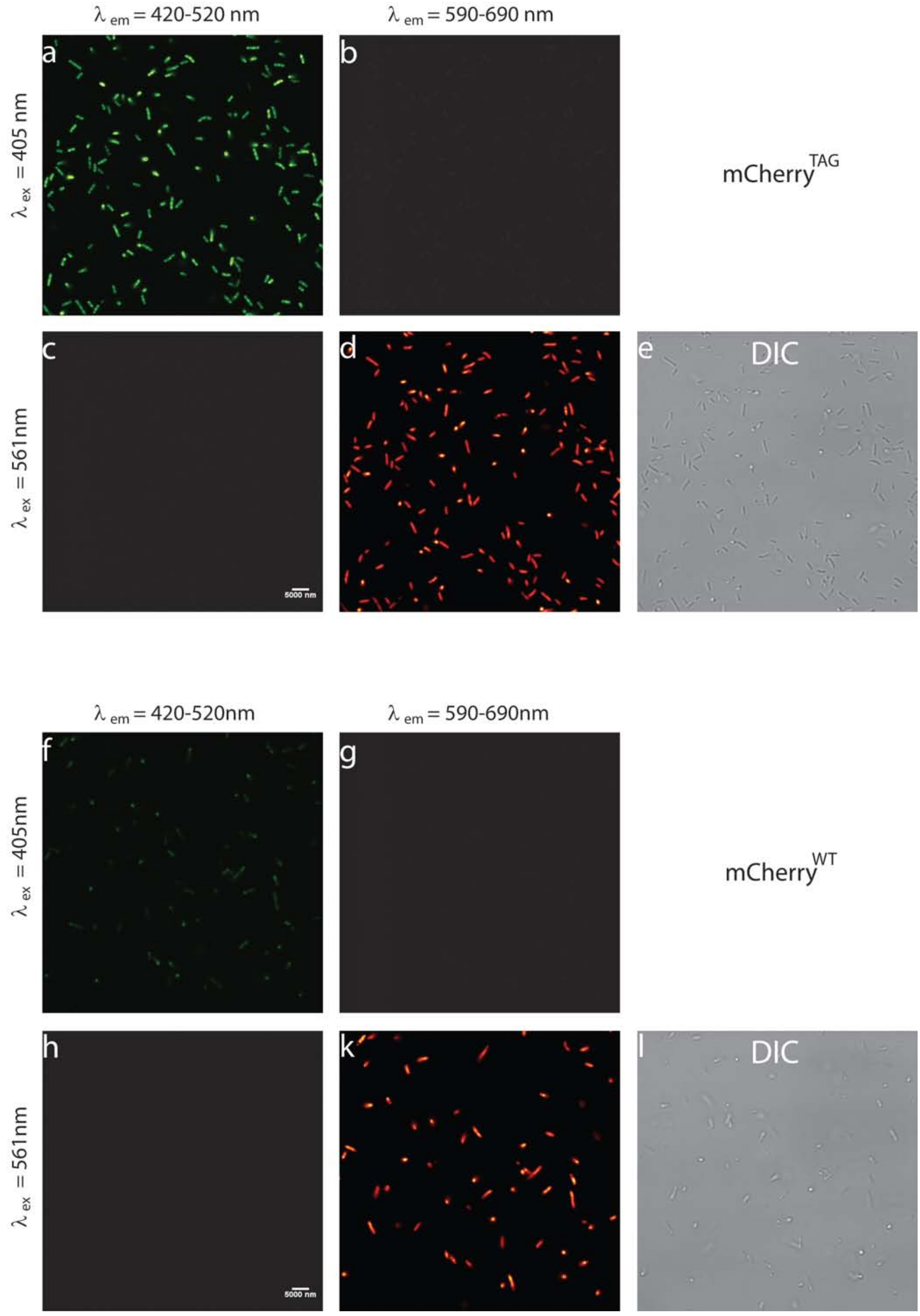
λ em = 420-520 nm
λ em = 590-690 nm
λ ex = 405 nm
λ ex = 561nm
a
b
c
d
e
DIC
mCherryTAG
5000 nm
λ em = 420-520nm
λ em = 590-690nm
λ ex = 405nm
λ ex = 561nm
f
g
h
k
l
DIC
mCherryWT
5000 nm

**Figure S4:** For fluorescence imaging of fluorogenic coumarin azide **3**, 5 ml $tRNA^{pyl}$/$pylRS^{AF}$ containing *E. coli* cultures expressing either $GFP^{TAG\rightarrow\mathbf{1}}$ or $GFP^{WT}$ were harvested after 4-6 h of induction and incubation with **1**, washed 2x with 12 ml PBS, resuspended in 12 ml PBS and incubated for 1 h at 4°C in the dark, and washed another 2x with 12 ml PBS. Cells were pelleted and resuspended in 3 ml PBS ($OD_{600}$ ~2-3) and then incubated with 50 µM **3** at 37˚C shaking in the dark. Cells were harvested after 3-4 h, washed two times with 1.5 ml PBS, resuspended in 1.5 ml PBS and incubated for 1 h at 4˚C in the dark, and washed another two times with 1.5 ml PBS before cells were allowed to settle on a coverslip. Cells were then mounted on Leica SP5 microscope employing a 1.4NA 63x oil objective (Leica, Mannheim, Germany). Images containing 512*512 pixels were acquired at a scan speed of 400 Hz and a zoom factor of three yielding a final pixel size of 160.5 nm*160.5 nm. In addition to a DIC image (e, l) the sample was excited using a blue diode laser operating at a wavelength of 405 nm, while simultaneously recording the fluorescence signal in two channels (blue/green= 415-470 nm and green= 520-540 nm). With cells expressing $GFP^{TAG\rightarrow\mathbf{1}}$, fluorescence in the blue/green channel originates from clicked coumarin (a), while fluorescence in the green channel originates from GFP (b). Please note that GFP can also be directly excited at 405 nm and fluorescence can also occur via energy transfer from clicked **3** to the GFP chromophore. In panel (c, d) the same emission channels were recorded during excitation with an argon ion laser operating at λ= 488 nm, which only excites GFP. As a control experiment the same staining and imaging procedure was repeated with cells expressing $GFP^{WT}$. In this construct, the synthetase and tRNA are still active in recognizing **1**, but wild type GFP contains no amber codon that allows incorporation of **1** and only background fluorescence is visible in (f). Since $GFP^{WT}$ naturally expresses better than the amber suppressed $GFP^{TAG\rightarrow\mathbf{1}}$ a stronger GFP fluorescence is observed in (k) compared to (d). Scale bar= 5 µm. In contrast to the mCherry experiments shown in Figure S3 and the main text, the similar spectral properties of GFP and **3** complicate high contrast image separation, and due to direct excitation and/or FRET the difference between (a) and (f) is seemingly less than in the mCherry experiments in Figure S3. However, this verifies that compound **3** ligated in vivo (see also Figure S5). The images in (a) and (f) were smoothed with a median 3*3 kernel and contrast/brightness were adjusted in the blue/green for image clarity.

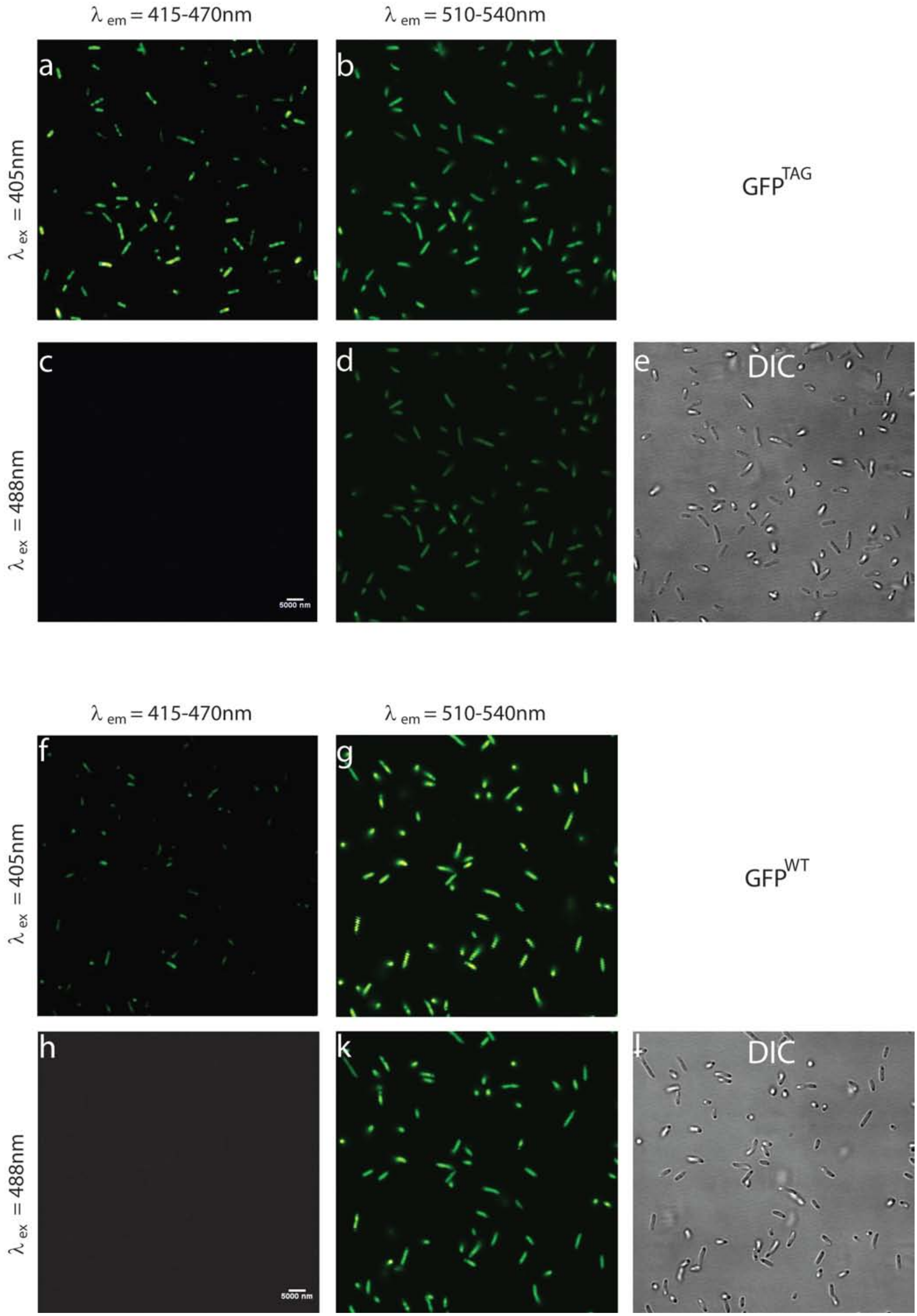

λ em = 415-470nm
λ em = 510-540nm
λ ex = 405nm
λ ex = 488nm
a
b
c
d
e
DIC
GFP^TAG
5000 nm
λ em = 415-470nm
λ em = 510-540nm
λ ex = 405nm
λ ex = 488nm
f
g
h
k
l
DIC
GFP^WT
5000 nm

**Figure S5:** Cultures used for imaging in Figure S4 were also analysed by SDS PAGE gels. After adding **3** to cultures expressing $GFP^{TAG\rightarrow\mathbf{1}}$ and $GFP^{WT}$ small samples were taken at the indicated time points and diluted to 1.5 ml PBS, washed 2x with 1.5 ml PBS, incubated at 4°C in the dark, washed another 2x with 1.5 ml PBS and then loaded on a SDS PAGE gel for whole cell lysate analysis. The gel was analysed for fluorescence using a commercially available gel documentation system (Alpha Innotech, CA) by exciting the sample at $\lambda$= 365 nm and detecting the emission with an ethidiumbromide filter setting. Already after 15 min $GFP^{TAG\rightarrow\mathbf{1}}$ (the GFP running height is indicated with an arrow) labelled with **3** is present, confirming the results from the imaging experiment that labelling of $GFP^{TAG\rightarrow\mathbf{1}}$ indeed occurred. The SDS PAGE gel indicates that in addition to GFP another larger protein is highly expressed, which is likely pylRS (MW 50.8 kDa). Due to the high expression a faint fluorescence of those bands (likely due to non-specific binding) is also visible in the fluorescence scan.

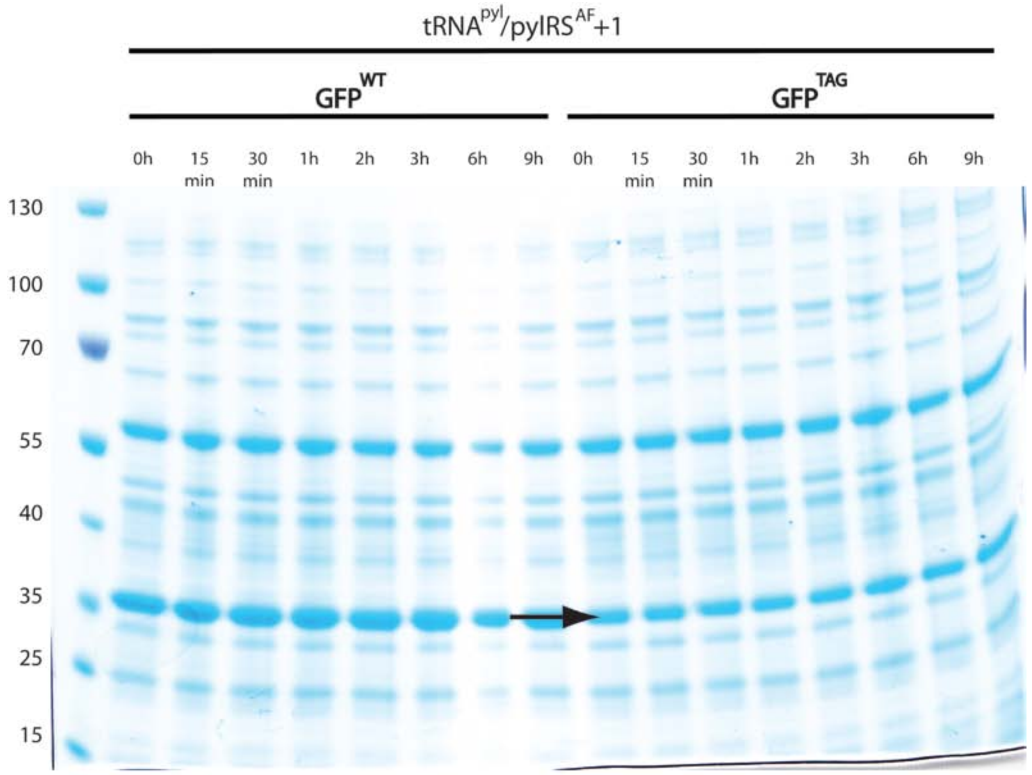


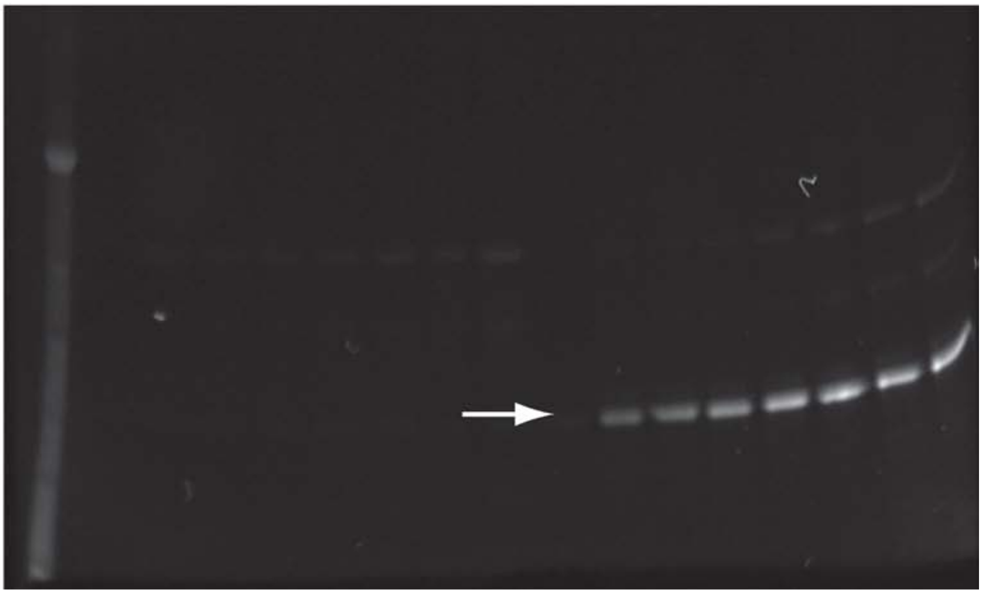

**Body text references:**

G. V. Los, L. P. Encell, M. G. McDougall, D. D. Hartzell, N. Karassina, C. Zimprich, M. G. Wood, R. Learish R, R. F. Ohana, M. Urh, D. Simpson, J. Mendez, K. Zimmerman, P. Otto, G. Vidugiris, J. Zhu, A. Darzins, D. H. Klaubert, R. F. Bulleit, K. V. Wood *ACS Chem. Biol.* **2008**, *3*, 373-382.

**Supplementary references:**

[1] T. S. Young, I. Ahmad, J. A. Yin, P. G. Schultz, *J. Mol. Biol.* **2010**, *395*, 361-374.
[2] E. A. Lemke, Y. Gambin, V. Vandelinder, E. M. Brustad, H. W. Liu, P. G. Schultz, A. Groisman, A. A. Deniz, *J. Am. Chem. Soc.* **2009**, *131*, 13610-13612.
[3] B. K. Muller, E. Zaychikov, C. Brauchle, D. C. Lamb, *Biophys. J.* **2005**, *89*, 3508-3522.
[4] a) A. B. Neef, C. Schultz, *Angew. Chem. Int. Ed. Engl.* **2009**, *48*, 1498-1500; b) *Angew. Chem.* **2009**, *121*, 1526-1529.
[5] C. B. Reese, A. Shaw, *J. Chem. Soc. Perkin Trans. 1* **1975**, 2422-2435.
[6] C. B. Reese, A. Shaw, *Chem. Commun.* **1970**, 1172-1173.
[7] K. Sivakumar, F. Xie, B. M. Cash, S. Long, H. N. Barnhill, Q. Wang, *Org. Lett.* **2004**, *6*, 4603-4606.
